# Altered Electroencephalographic Activity Associated with Changes in the Sleep-Wakefulness Cycle of C57BL/6J Mice in Response to a Photoperiod Shortening

**DOI:** 10.3389/fnbeh.2016.00168

**Published:** 2016-08-31

**Authors:** Stanislav V. Rozov, Janneke C. Zant, Kestutis Gurevicius, Tarja Porkka-Heiskanen, Pertti Panula

**Affiliations:** ^1^Department of Anatomy, Faculty of Medicine, Neuroscience Center, University of HelsinkiHelsinki, Finland; ^2^Department of Physiology, Faculty of Medicine, University of HelsinkiHelsinki, Finland; ^3^A. I. Virtanen Institute for Molecular Sciences, University of Eastern FinlandKuopio, Finland

**Keywords:** circadian, entrainment, sleep, desynchrony, wakefulness

## Abstract

**Aim:** Under natural conditions diurnal rhythms of biological processes of the organism are synchronized with each other and to the environmental changes by means of the circadian system. Disturbances of the latter affect hormonal levels, sleep-wakefulness cycle and cognitive performance. To study mechanisms of such perturbations animal models subjected to artificial photoperiods are often used. The goal of current study was to understand the effects of circadian rhythm disruption, caused by a short light-dark cycle regime, on activity of the cerebral cortex in rodents.

**Methods:** We used electroencephalogram to assess the distribution of vigilance states, perform spectral analysis, and estimate the homeostatic sleep drive. In addition, we analyzed spontaneous locomotion of C57BL/6J mice under symmetric, 22-, 21-, and 20-h-long light–dark cycles using video recording and tracking methods.

**Results and Conclusions:** We found that shortening of photoperiod caused a significant increase of slow wave activity during non-rapid eye movement sleep suggesting an elevation of sleep pressure under such conditions. While the rhythm of spontaneous locomotion was completely entrained by all light–dark cycles tested, periodic changes in the power of the θ- and γ-frequency ranges during wakefulness gradually disappeared under 22- and 21-h-long light–dark cycles. This was associated with a significant increase in the θ–γ phase-amplitude coupling during wakefulness. Our results thus provide deeper understanding of the mechanisms underlying the impairment of learning and memory retention, which is associated with disturbed circadian regulation.

## Introduction

The ability to adjust to periodic environmental changes is one of the key properties of the circadian system. To fulfill this function the central circadian oscillator sets the pace for a large variety of biological processes ranging from synaptic plasticity up to higher cognitive functions (Barnes et al., 1977; Gerstner and Yin, 2010). In vertebrates the master circadian oscillator resides in the suprachiasmatic nuclei (SCN) of the hypothalamus (Moore and Eichler, 1972). The importance of the circadian regulation can be clearly seen when the clock work is altered by distorted light-dark cycles, like excessively long illumination periods during 24 h, rotating shift work (Knutsson, 2003; Boivin et al., 2007) or trans-meridian flights (Cho, 2001). The common features of these light-dark regimes are advances or delays of a photoperiodic cycle that cannot be entrained by the circadian clock.

To characterize changes of the circadian regulation under such conditions studies on animals, subjected to light-dark cycles beyond the entrainment range of the circadian clock, have been performed. These experimental protocols non-invasively disrupt normal functioning of the circadian system, which appears as an altered electrophysiological activity of the neurons in the SCN (Houben et al., 2014), expression of core clock genes *Per1* and *Bmal1* in this brain region (de la Iglesia et al., 2004) and production of melatonin, which serves as a hormonal arm of the oscillator (Schwartz et al., 2009). It is accompanied by changes in a rest-activity cycle (Tribukait, 1956; Campuzano et al., 1998; Udo et al., 2004), leptin and insulin secretion (Karatsoreos et al., 2011) and functioning of the immune system (Phillips et al., 2015). Besides the effects on behavior and metabolism, inability to entrain to short cycles affects memory retention (Devan et al., 2001; Loh et al., 2010; Karatsoreos et al., 2011) and the sleep-wakefulness cycle. In the latter case, several studies on humans (Wyatt et al., 1999) and rodents (Laakso et al., 1995; Cambras et al., 2007; Lee et al., 2009) utilizing symmetric 10/10 or 11/11 light-dark (LD) schedules, reported desynchronization between the locomotor activity and NREM-sleep that primarily followed LD cycle and REM-sleep and body temperature that were adhered to the internal circadian rhythm.

Noteworthy, although some of these studies utilized electroencephalographic (EEG) recording, this method was mainly used to discriminate between sleep and wakefulness states, whereas little attention was paid to the quantitative analysis of changes in the brain electrophysiological activity. At the same time, many aspects of cortical activity, including δ- and θ-waves that are known as correlates of the homeostatic sleep drive and attention, respectively, undergo prominent diurnal changes (Daan et al., 1984; Welsh et al., 1985).

Therefore, the aim of this study was to examine the effects of circadian rhythm disruption, caused by a short light-dark cycle regime, on cortical activity which was assessed by means of quantitative electroencephalographic analysis. To draw the parallel with previous studies, we first quantitatively examined changes in the characteristics of spontaneous locomotion, wakefulness, NREM-sleep and REM-sleep stages in response to an altered zeitgeber rhythm. We further used EEG to assess the distribution of vigilance states, track the dynamical changes of individual frequency components, estimate the homeostatic sleep drive and perform the state-specific exploratory analysis of the phase-amplitude coupling (PAC).

## Materials and methods

### Animals

Seven- to 10-week-old male C57BL/6J mice were supplied by Charles River Laboratories (Chatillon-sur-Chalaronne, France). Animals were individually housed at 21 ± 2°C, with free access to standard food pellets (Scanbur, Sollentuna, Sweden) and water *ad libitum*. Mice were kept individually for 2 weeks before surgery. Before the experiments, we implanted the animals with electrodes for EEG, electromyography (EMG), and microdialysis probe. At baseline, mice were maintained at a reversed symmetric 12-h light–12-h dark (LD 12/12) cycle (lights off at 8:30, < 0.5 lux; lights on at 20:30; cool white light at 170 lux at the bottom of the cage). During the experiment, animals were sequentially subjected to LD 11/11, dark–dark (DD), LD 10.5/10.5, and LD 10/10 regimes for 14, 7, 14, and 14 astronomic days respectively. All experiments were performed according to the Finnish Act on the Use of Animals for Experimental Purposes, and approved by the Animal Experiment Committee of the State Provincial Office of Southern Finland, and the European Communities Council Directive of 24 November 1986 (86/609/EEC). We also adhered to the guidelines laid down by the National Institutes of Health (NIH) in the United States regarding the care and use of animals for experimental procedures. From the total number of animals used in this study (*n* = 8), we excluded one due to an equipment failure. Altogether, seven mice were used for behavioral analysis and six underwent EEG analysis.

### Surgery

Animals were operated on general anesthesia, induced by ketamine (Ketalar^©^, Pfizer Animal Health, New York, USA; 75 mg kg^−1^, i.p.) combined with medetomidine (Domitor^©^, Pfizer Animal Health; 1 mg kg^−1^, i.p.). After exposure, skull bones were cleaned and disinfected. Two gold-coated screws were installed into the skull for the frontoparietal epidural recording of EEG. Electrodes for EMG were placed into the neck musculature. The guide cannula (CMA 7 Guide, CMA/Microdialysis, Solna, Sweden) was implanted into the posterior part of the hypothalamus at the following stereotaxic coordinates (relative to bregma): anterior, −2.5 mm; lateral, +0.5 mm; vertical, −4.4 mm (Paxinos and Franklin, 2004); and 1 mm above the tuberomamillary nuclei. The electrodes, guide cannula, and supporting screws were secured to the skull using dental cement (Candulor, Wangen, Germany). To recover from anaesthesia, mice were injected with atipamezole hydrochloride (Antisedane^©^, Pfizer Animal Health; 0.5 mg kg^−1^, s.c.) and given the analgesic buprenorphine (Temgesic^©^, Reckitt Benckiser, Slough, UK; 0.1 mg kg^−1^ s.c.) two times during first 24 h after surgery. To ensure that animals recovered from surgery, their spontaneous locomotion was compared to that prior to surgery, as well as overall appearance, food and water consumption and absence of symptoms of distress were examined. EEG recording started after 1-week-long recovery period. Two or three days after the start of EEG/EMG recording, a microdialysis probe (CMA 7, 1-mm membrane; CMA/Microdialysis) was implanted in the posterior hypothalamus. The stereotaxic coordinates of the probe tip (relative to bregma) were: anterior, −2.14 to −3.07 mm; lateral, +0.5 mm; and vertical, −5.4 mm (Paxinos and Franklin, 2004). The probe was connected to a sample collection system, followed by continuous perfusion (1 μL min-1) with artificial cerebrospinal fluid (147 mM NaCl, 3 mM KCl, 1.2 mM CaCl_2_, and 1 mM MgCl_2_; reagents purchased from Merck, Whitehouse Station, NJ, USA). The microdialysis analysis was performed for another study and is not further considered here.

### Video recording and tracking

Each cage was equipped with a CAMZWMBLAH2N video camera (Velleman, Belgium) combined with an infrared light source. The video stream was captured and recorded continuously using GeoVision Surveillance software (GeoVision, Taiwan) beginning 5 days before the surgery continuing until the end of the experiment. The recorded video data were converted and prepared for tracking by VirtualDub 1.9.2 (www.virtualdub.org), and tracking was then performed with the EthoVision 3.1 software package (Noldus Software, Netherlands). The distance moved in a cage was calculated in 1-min time bins.

### EEG/EMG recording and analysis of the distribution of vigilance states

EEG/EMG recording began 5–6 days after surgery and continued throughout the duration of the experiment. The EEG and EMG signals were amplified (gain 10,000), filtered (high pass, 0.3 Hz; band stop, 50 Hz; and low pass, 100 Hz) and sampled at 200 Hz using Spike2 software (version 6, Cambridge Electronic Devices, Cambridge, UK). To remove the low-frequency artifacts, EEG signals were filtered using a high pass filter (lower cut-off frequency: 0.5 Hz) with Spike2 software. The EEG recordings were scored semi-automatically using the algorithm developed by Rytkönen et al. (2011) and further manually verified for the absence of artifacts. Scoring was performed on 4-s epochs for wakefulness, NREM, and REM sleep. The vigilance states were distinguished as follows: wakefulness was defined based on desynchronized EEG activity accompanied by dampened slow wave (SWA; 1–4 Hz) oscillations; NREM sleep was defined as high amplitude slow wave activity (1–4 Hz) in EEG and low amplitude or absent in EMG; and REM sleep was defined as regular high θ (7–9.5 Hz) activity in EEG and decreased or absent in EMG. The resulting data were used to derive the average amount and number of episodes of wakefulness, NREM, and REM sleep, as well as state-specific power spectra. Power spectra were computed using fast Fourier transform (FFT) with the following parameters: FFT size, 512; epoch, 4 s Hann window; frequency range, 0–50 Hz. Power spectra were normalized using the division of values at each vigilance state per median total power for the corresponding day. The normalized values were averaged across the last 2 days of the corresponding light–dark stage of the experiment for each individual animal and used for statistical evaluation.

### Phase-amplitude coupling (PAC) analysis

Non-processed EEG data from the last 2 days of each phase of the experiment were exported from Spike2 to the Matlab format and filtered with a set of finite-response band-pass filters (step: 1 Hz; window width: 1 Hz; filter length: 1500 time points) ranging from 2 to 100 Hz using MatLab 7.5 (MathWorks, Nattick, USA). We then subjected filtered data to a Hilbert transform to obtain instantaneous phase and power values. PAC was computed over one complete cycle in 1-min bins, separately for wakefulness, NREM, and REM sleep states using the following equation (Canolty et al., 2006):

$$PAC=\left|\frac{\sum_{t=1}^{n}a^{t}e^{i\varphi t}}{n}\right|,$$

where *t* – time point, *a* – power of modulated frequency at time point *t*, *i* – imaginary operator, φ – phase angle in radians of a carrier frequency at time point *t*, and *n* – total number of time points.

The normalized PAC values (PACz) were computed from the empirical distribution of PAC values created by a random reordering power time series relative to a phase time series followed by the calculation of the re-sampled PAC (Cohen, 2014). Next, we computed matrices of median PACz values frequency pair-wise for light and dark periods separately. We used paired *t*-tests to compare PACz values between different conditions. To correct for multiple comparisons, we thresholded resulting matrices of *t*-values to retain only those values that exceeded *p* = 0.01, excluding the remainder from further analysis. Integrated *t*-values were computed for any contiguous array (cluster) of suprathreshold values. We tested these values for significance against the distribution of integrated *t*-values of clusters acquired through the permutation-based generation of sets of matrices of *t*-values, followed by thresholding as described above. We considered clusters that exceeded *p* = 0.05 as significant. A detailed description of this procedure can be found in Cohen (2014).

### Statistical analyses

Linear mixed effect model (LMM) analysis was performed using the *nlme* library (*nlme: Linear and Nonlinear Mixed Effects Models. R software* 2013) for the R software program (Pinheiro et al., 2016). The model was specified as follows:

$$\begin{array}{l} y_{ijk}=m+\alpha_{j}+\beta_{k}+\gamma_{jk}+b_{i}+\varepsilon_{ijk}, \end{array}$$

where *y*_*ijk*_ – response vector of the *ith* subject at period length *j* during phase *k*; *m* – grand mean; α_*j*_ – main effect of the period length, where *j* = 1…4 (locomotor activity during either LD12/12, LD11/11, LD10.5/10.5 or LD10/10) or *j* = 1…3 (either LD12/12, LD11/11 or LD10.5/10.5) – number of periods tested; β_*k*_ – main effect of a phase, where *k* = 1, 2 – photophase or scotophase; γ_*jk*_ – period-phase interaction; *b*_*i*_ – random effect of the subject; *i* = 1…7 – subject tested; ε_*ijk*_ – random error; and *b*_*i*_~*N*(0,σ^2^_*b*_), ε = *N*(0,σ^2^), σ^2^ – unknown variance.

**Figure 1:**
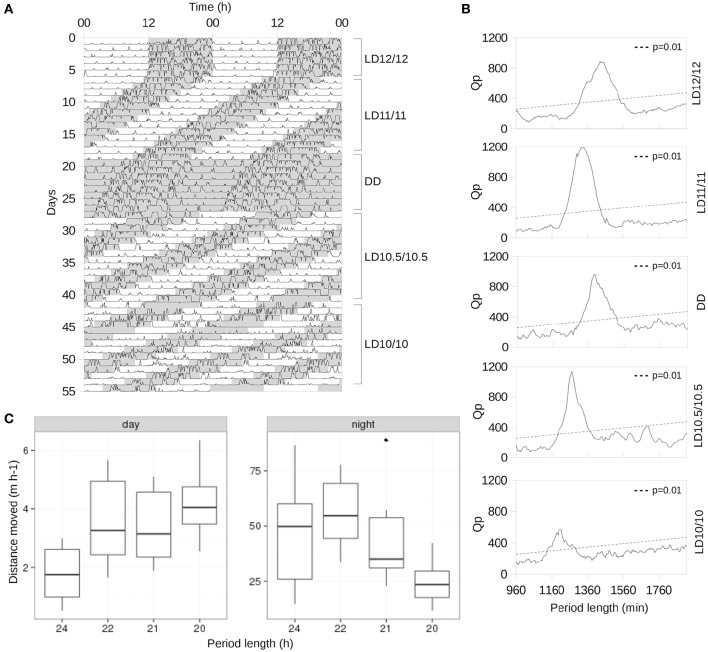
**Changes in the spontaneous locomotor activity in response to short photoperiods. (A)** Representative actogram of an animal, single-housed under LD12/12 for the first 7 days, then 14 days at LD11/11, then released to DD for 7 days as the washout period. Subsequently, the animal was subjected to an LD10.5/10.5 cycle for 14 days and, finally, to LD10/10 for 14 days. Shaded areas represent dark periods. **(B)** χ^2^-periodograms of the animal shown in **(A)** computed for all light–dark schedules. Dashed line represents Qp values at *p* = 0.01. **(C)** Boxplot of average distances moved per hour under different period lengths during the day and night, respectively: solid horizontal bar represents median values, while the upper and lower limits of the box represent the first and third quartiles.

As the fixed effect, we chose the period length and the phase of the day, and as the random effect we selected the effect of the individual specified as the intercept. Since the spontaneous locomotion of some animals exhibited an apparent non-linear response to a period shortening (Figure 1C, right panel), we decided to include a quadratic term in the model. The *p*-values were obtained using a likelihood ratio test (LRT) of the full model with the factors “period length,” “phase,” and their interaction against the models without either term. The confidence intervals of the estimated parameters, the overall fit of the data set to the model, tests for influential data points as well as assumptions of normality and the homogeneity of the residuals were also examined.

To quantitatively assess the period length of the locomotor activity under different light–dark cycles, we used the non-parametric χ^2^ periodogram (Sokolove and Bushell, 1978), with the significance level set at *p* = 0.01.

We computed the power spectra for one full cycle of recording and compared these using the means of multiple paired *t*-tests, followed by a correction for multiple comparisons as described for the PACz values.

Statistical evaluation of the periodical properties of the θ- and γ-frequency band time series during wakefulness under different light–dark cycles was performed by means of cosinor analysis (Bingham et al., 1982) using Matlab 7.5. We filtered the raw EEG recordings from the last complete cycle of a corresponding light–dark regime as described above to derive a time series of PSD for the θ- and γ-frequency bands. These PSD time series were normalized subject-wise per median PSD for the corresponding frequency band computed under LD12/12 regime. The normalized time series were grouped into 10-min bins, and fitted with the cosine function:

$$\begin{array}{l} y_{i}=M+A^{*}cos\left(\omega t_{i}+\theta \right)+\varepsilon_{i}, \end{array}$$

where *y*_*i*_ – response vector of the *i*th measurement at time *t*, *M* – mesor, A – amplitude, ω – angular frequency (degrees hour-1), *t*_*i*_ – time of the *i*th measurement, θ – phase (degrees), ε_*i*_ = (0, σ^2^), and σ^2^ – unknown variance.

This function was linearized for all parameters to the form:

$$\begin{array}{l} y_{i}=M+\beta x_{i}+\gamma z_{i}+\varepsilon_{i,} \end{array}$$

where *y*_*i*_ – response vector of the *i*th measurement at time *t*, *M* – mesor; β = *A* ^*^ cos θ, *x*_*i*_ = cos ω*t*_*i*_, γ = −*A* ^*^ sin θ, *z*_*i*_ = sin ω*t*_*i*_, ε_*i*_ = (0, σ^2^), A – amplitude, ω – angular frequency (degrees hour-1), *t*_*i*_ – time of the *i*th measurement, and σ^2^ – unknown variance.

To estimate the group-level phase, amplitude, and mesor values, determine their significance, and compare different light–dark conditions, we completed a group cosinor test based on previously conducted individual cosinor analyses.

According to the two-process model (Daan et al., 1984), slow wave activity (SWA) exponentially decays during NREM sleep. To quantify this decay, we used a function proposed in the original publication (Daan et al., 1984):

$$\begin{array}{l} SWA_{t}=SWA_{0}{\;}^{*}\;e^{-t/\tau }+SWA_{inf}, \end{array}$$

where *SWA*_*t*_ – estimate of SWA at time *t*, SWA_0_ – SWA at time 0 (first incidence of NREM sleep), *t* – time at which the measurement was conducted, τ – decay constant, and *SWA*_*inf*_ – asymptotic SWA.

EEG data were filtered as described above, and a 1–3-Hz frequency window was selected to examine changes in SWA. Data were binned NREM-state specifically to 10-min bins and normalized subject-wise per median SWA over the photophase. We used GraphPad Prism software (GraphPad Inc., USA) to fit and statistically evaluate the data acquired under different illumination conditions. We considered the estimated parameters *SWA*_0_, τ, and *SWA*_*inf*_ significant at *p* < 0.01 (*F*-test).

To assess sleep pressure under different circadian regimes, we characterized changes in SWA buildup upon transition from wake to NREM sleep by means of the following logistic regression model:

$$\begin{array}{l} y_{t}=min+\left(max-min\right)/\left(1+10^{\left(logt50-1\right)\;*\;Slope}\right), \end{array}$$

where *y*_*t*_ – estimate of SWA at time *t, min* – baseline SWA, *max* – maximal SWA reached within 60 s after the transition occurred, *log*_*t*50_ – time at which 50% SWA is reached, and *Slope* – is the steepness of the change in SWA.

We processed and analyzed EEG data as described above, but set the time bin to 2 s. We considered the estimated parameters *min*, *max*, *logt50*, and *Slope* significant at *p* < 0.01 (*F*-test).

## Results

Our analysis of spontaneous locomotion revealed its stable entrainment to LD12/12, LD11/11, and LD10.5/10.5 cycles (Figures 1A,B) among all animals tested. Under LD10/10 spontaneous locomotion was largely suppressed (Figure 1C), and redistributed between day and night making activity periods less prominent compared to other regimes (Figures 1B,C). The shortening of the circadian period was accompanied by a gradual linear increase in the average distance moved during the daytime (*p* = 0.004, LMM followed by LRT, Figure 1C, left panel) and a slight increase (*p* < 0.001, LMM followed by LRT) followed by a linear decrease at night (*p* = 0.008, LMM followed by LRT, Figure 1C, right panel).

**Figure 2:**
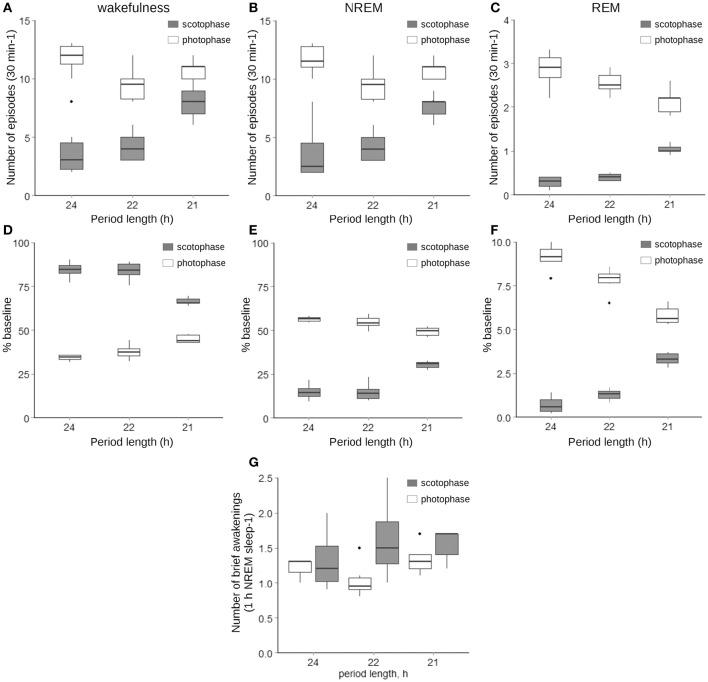
**Number and lengths of wakefulness (A,D), NREM (B,E), and REM sleep (C,F) episodes as well as the number of brief awakenings per h of NREM sleep (G) under LD12/12, LD11/11, and LD10.5/10.5 cycles**. Gray boxes represent values acquired during the scotophase, while white—during photophase; the thick line inside each box indicates the median of the sample, the lower and upper boundaries represent the 25th and 75th percentiles of the sample, the whiskers corresponds to the 5th and 95th percentiles of the sample, and the individual black dots indicate values that exceed the 5th and 95th percentiles. The interaction between the period length and the phase of the cycle was significant at *p* < 0.001 for all variables represented (LMM followed by LRT, *n* = 5–6).

Changes in the animals' activity toward an equal distribution across the cycle were mirrored by a rearrangement of the sleep–wake architecture, where both the number of fragments (Figures 2A–C) and percentage of total time spent in either vigilance state (Figures 2D–F), show similar tendencies. The changes were highly dependent on the interaction between the phase of the light–dark cycle and the period length (*p* < 0.001, LMM followed by LRT). The number of brief awakenings normalized per percentage of NREM sleep remained similar independent of the period length (Figure 2G).

**Figure 3:**
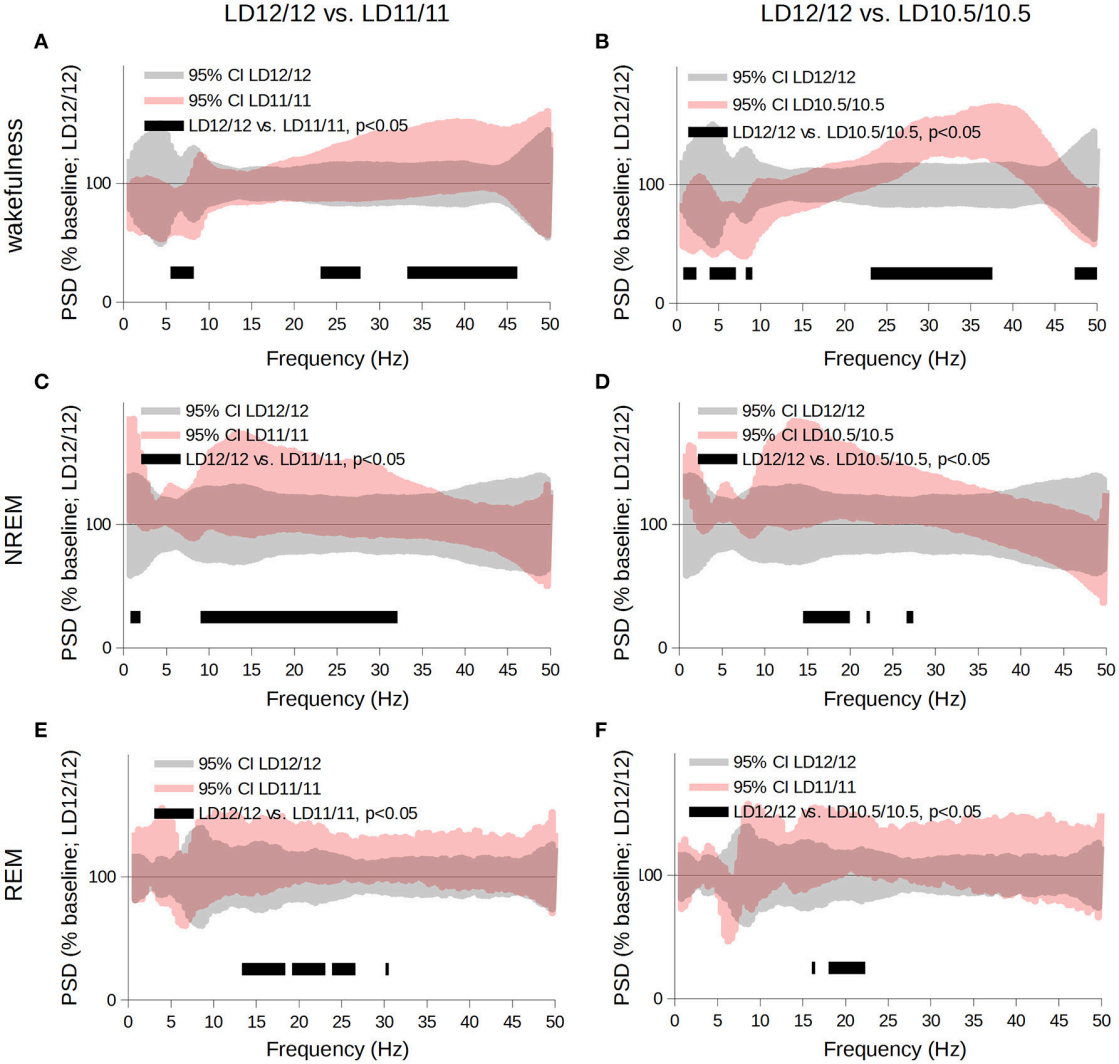
**Comparison of the power spectra acquired for wakefulness (A,B), NREM- (C,D), and REM- sleep (E,F) stages throughout the complete circadian cycle 24- (gray area), 22- or 21-h-long (pink area)**. Data are shown as the 95% confidence intervals of the means expressed as a percent of the baseline (LD 12/12). We used the multiple paired *t*-tests for statistical evaluation followed by correction for multiple comparisons using the cluster-based approach; black bars correspond to clusters different from baseline at *p* < 0.05.

The comparative analysis of the frequency spectra revealed that, during the wakefulness state, the power of the θ frequency gradually decreased, whereas the power of the γ-frequency band increased (Figures 3A,B, *p* < 0.05, Student's *t*-test); during NREM sleep, SWA and the power of the α- and β-frequency ranges increased compared to the LD 12/12 regime (Figures 3C,D, *p* < 0.05, Student's *t*-test). During REM sleep, PSD of the α- and β- frequency ranges increased compared to the LD12/12 regime (Figures 3E,F, *p* < 0.05, Student's *t*-test). Next, we performed state-specific analyses of the periodograms of the frequency bands mentioned above.

**Figure 4:**
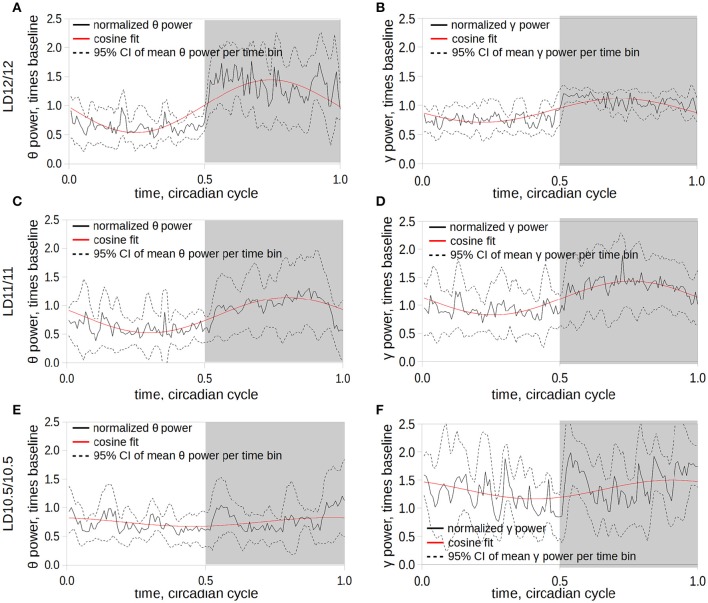
**Dynamics of the changes in power of θ- (A,C,E) and γ- (B,D,F) frequency ranges under LD12/12 (A,B), LD11/11 (C,D), and LD10.5/10.5 (E,F)**. The time series of the θ-power were normalized subject-wise per median value of θ-power under LD12/12 and grouped in 10-min bins. Solid black line corresponds to the mean power value computed at the 10-min time bin; dashed lines represent 95% confidence intervals of the mean at a given time; solid red line represents the fit of the power time series using a cosine curve; and gray area corresponds to the dark period of the cycle.

The daily rhythms of power of θ- and γ-frequency bands during wakefulness were prominent under LD12/12 (Figures 4A,B) and LD11/11 (Figures 4C,D) conditions, and completely disappeared under the LD10.5/10.5 regime (Figures 4E,F, *p* < 0.05, group-cosinor analysis followed by *F*-test). This was accompanied by an elevation in the phase angle variation of the γ band compared to LD12/12 schedule (Figure 4F).

**Figure 5:**
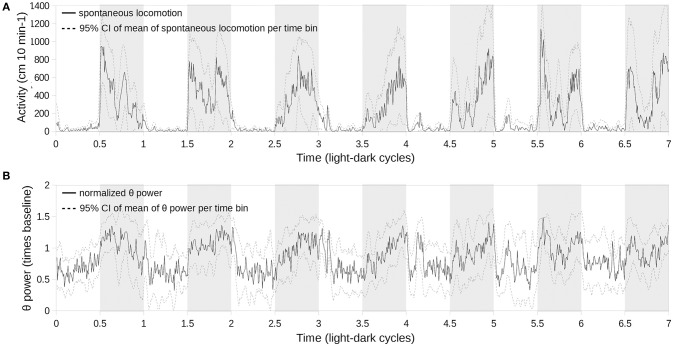
**Concomitant evolution of spontaneous locomotion (A) and θ-power (B) periodicity upon switching from constant darkness to LD11/11 (time point 0) over the subsequent 7 days**. θ-power was normalized subject-wise per median θ-power during the first cycle. Solid lines correspond to the mean values of locomotor activity and θ-power, respectively, sampled at 10-min bins; light gray dashed lines represent 95% confidence intervals for the corresponding mean values; and shaded areas represent the scotophase.

Finding a complete disappearance of the diurnal periodicity of the θ- and γ-frequency bands led us to test if this effect is caused by an inability of the oscillator to entrain to a short cycle or by other factors. Therefore, we followed the concomitant changes in spontaneous locomotion and the θ band for over seven light–dark cycles starting from the switch from constant darkness when all animals exhibit a free-running periodicity to the LD10.5/10.5 regime (Figure 5). After 5–6 days, the θ-wave activity during wakefulness became aperiodic (Figure 5B) and corresponded to that seen in Figure 4, whereas spontaneous locomotion was entrained to the light–dark cycle (Figure 5A).

**Figure 6:**
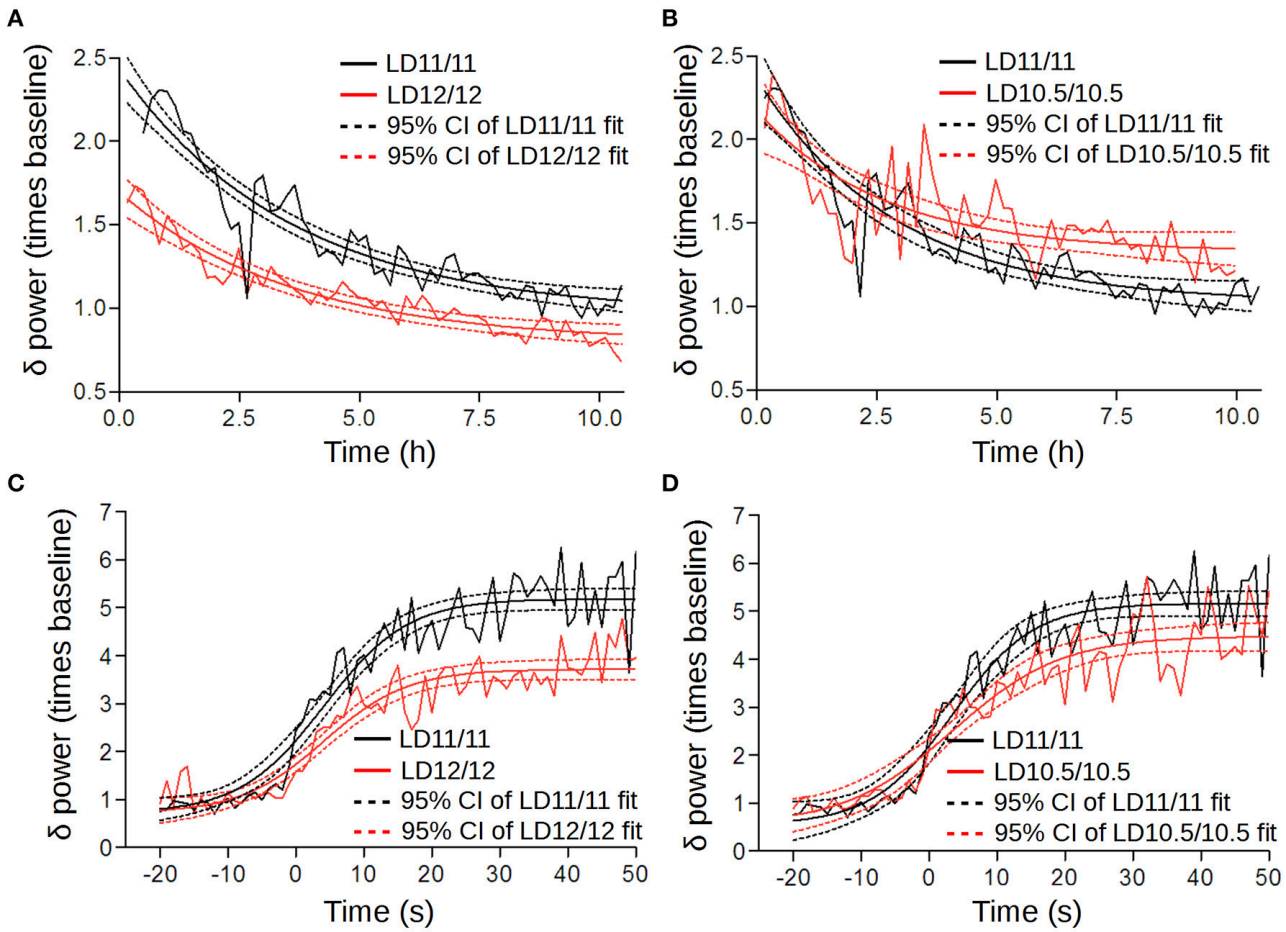
**Homeostatic regulation altered under different circadian cycles. (A,B)** The time series of SWA were normalized subject-wise per median NREM SWA value (power of the δ-frequency band) under LD12/12 and grouped in 10-min bins. These were then fitted by an exponential decay function (sleep phase of the two-process model) using a non-linear regression. Solid lines correspond to the fitted decay function; dashed lines represent the 95% confidence intervals. We found a significant elevation in the amplitude between LD12/12 (red) and LD 11/11 (black) **(A)**, whereas the rate of decay and low asymptote were similar under LD11/11 and LD 10.5/10.5 **(B)**. **(C,D)** Changes in SWA upon the wake-to-NREM transition under different light–dark cycles; zero point corresponds to the phase transition time. Black color corresponds to the LD11/11 cycle, red color represents the LD12/12 and LD10.5/10.5 cycles.

The power and decay rates of NREM SWA were significantly higher under short cycles compared to the LD12/12 regime [LD12/12 vs. LD11/11: Figure 6A; *F*_(2, 757)_ = 96.27, *p* < 0.001, *F*-test; LD11/11 vs. LD10.5/10.5; Figure 6B; *F*_(3, 645)_ = 10.03, *p* < 0.001, *F*-test; LD12/12 vs. LD10.5/10.5: *F*_(3, 647)_ = 128.9, *p* < 0.001, *F*-test], suggesting an elevated sleep pressure under short cycles. This was further confirmed by an analysis of the buildup rate of NREM SWA. Here, we found that the maximum power was reached 50 s after the wakefulness-to-NREM transition which was significantly higher under LD11/11 and LD10.5/10.5 compared to LD12/12 [LD12/12 vs. LD11/11: Figure 6C; *F*_(2, 1201)_ = 56.51, *p* < 0.001, *F*-test; LD11/11 vs. LD10.5/10.5: Figure 6D; *F*_(1, 775)_ = 59.26, *p* < 0.001, *F*-test; LD12/12 vs. LD10.5/10.5: *F*_(2, 775)_ = 19.56, *p* < 0.001, *F*-test).

**Figure 7:**
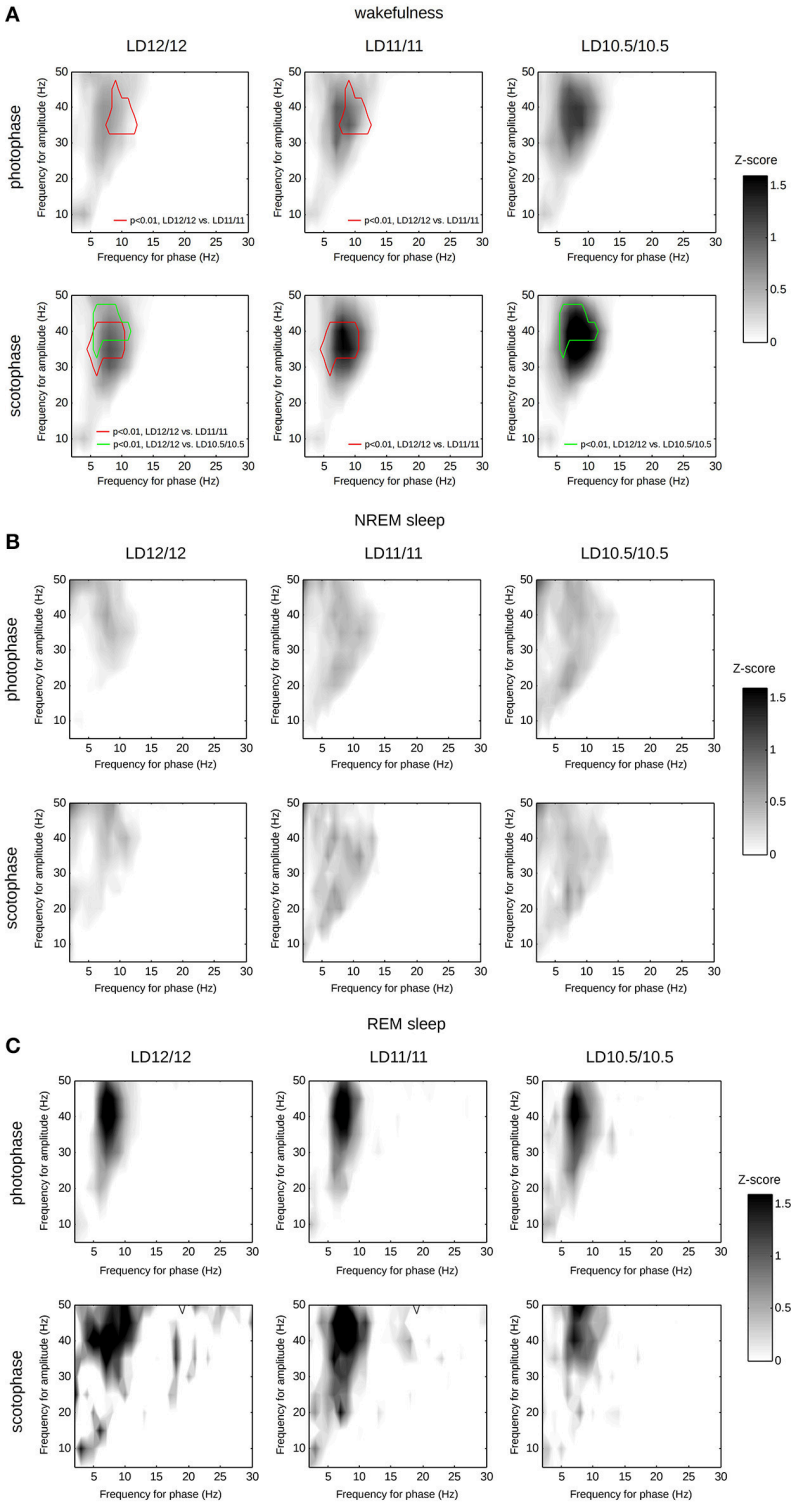
**Phase-amplitude coupling computed separately for wakefulness (A), NREM sleep (B), or REM sleep (C) over photophase or scotophase under different light–dark cycles**. Each heat-map represents the median PACz values computed frequency-wise. We compared the PACz matrices from the corresponding groups using paired *t*-tests for each frequency couple, followed by a clustering of *t*-values and the identification of statistically significant clusters using permutations. Red contour corresponds to a statistically significant cluster of *t*-values computed for LD12/12—LD11/11 comparison (*p* < 0.01, multiple *t*-tests, followed by permutation-based cluster thresholding); green contour corresponds to a statistically significant cluster of *t*-values computed for LD12/12—LD10.5/10.5 comparison.

The modulation of the γ-frequencies by θ-waves during wakefulness as well as concomitant changes of SWA, α- and β- bands during NREM sleep were previously described, including several proposed measurements for these interactions (Tort et al., 2009; Jirsa and Müller, 2013). Among others, PAC is best studied, and described for both wakefulness and NREM and REM states (Scheffzük et al., 2011). Therefore, we conducted an exploratory analysis of PAC across the 1–50-Hz frequency range for each vigilance state separately. We found a significant progressive elevation of PAC between the γ- and θ-frequency ranges during wakefulness upon shortening the diurnal light–dark period (Figure 7). This effect appeared during both the scotophase and photophase. We found no other significant changes in PAC, albeit our measurements likely underestimated the true differences (van Driel et al., 2015).

## Discussion

Our key findings include the following: (1) the disappearance of circadian changes at the θ- and γ-frequency ranges under LD10.5/10.5 regime during wakefulness; (2) a significant elevation of SWA during NREM sleep under LD11/11 and LD10.5/10.5; and (3) an increase in θ-γ PAC under LD11/11 and LD10.5/10.5 during wakefulness.

Under LD11/11 we observed a significant increase in SWA during photophase, which may be a sign of elevated sleep pressure. The wakefulness state was characterized by a strengthening of the θ-γ PAC compared to LD12/12. The spontaneous locomotion was completely entrained to the LD cycle. Although the activity maximum shifted from the beginning of the scotophase to mid-night, the shift was stable for more than 10 days (data not shown). Houben et al. (2014) recently reported similar results, whereby this shift of the locomotor activity peak was accompanied by increased activity in SCN during the first half of the scotophase. Furthermore, as the period shortened, SCN became active for a longer time during the dark period. In addition, several researchers previously reported a negative correlation in the multiunit recording of SCN activity and locomotion (Deboer et al., 2003; Nakamura et al., 2008; Houben et al., 2009). These are likely mutually inhibitory processes, since the suppression of SCN by tetrodotoxin leads to the induction of locomotion (Houben et al., 2014), whereas forced locomotion causes the suppression of activity among most neuronal sub-populations in SCN (Deboer et al., 2003; van Oosterhout et al., 2012). Interestingly, in rats, subjected to similar conditions, spontaneous locomotion exhibits two periodicities that have lengths 22 h and near-25 h and correspond to intrinsic circadian and light-driven pacemakers (Campuzano et al., 1998; de la Iglesia et al., 2004; Schwartz et al., 2009), whereas mouse locomotor activity becomes completely entrained to light-dark cycle.

In contrast to LD11/11, the transition from the DD to LD10.5/10.5 cycle led to a profound redistribution of the wakefulness, NREM and REM sleep stages toward an aperiodic state, although locomotion still entrained to the light–dark cycle. In terms of the distribution of the sleep–wake states, our results are in good agreement with studies of sleep distribution in rats with the SCN lesion (Coindet et al., 1975) and mice, subjected to a LD10/10 cycle (Phillips et al., 2015). Besides that the latter study reported flattened pattern of the SWA throughout a short LD cycle, which matches well with our data. An examination of concomitant changes of locomotor activity and changes in the power of the θ-frequency revealed a gradual shift in the maximum values for both parameters toward the end of the scotophase, followed by the disappearance of circadian periodicity at the θ-frequency at day 5 under the LD10.5/10.5 cycle. Examining the directionality of SCN activity changes in response to shortening light–dark cycles (Houben et al., 2014), its anti-phase occurrence with locomotor activity, and the high positive correlation of locomotor activity to PSD θ- and γ-frequency bands during wakefulness, we speculate that the changes in the θ frequency may result from the inability of the oscillator to entrain the light–dark cycle. This culminates in its suppression or splitting by light in a manner similar to a previous study (Nakamura et al., 2002, 2005).

In order to explain the observed alteration in the θ-frequency circadian rhythm, we assume a primarily hippocampal origin. However, we are aware that the current experimental setup utilizing only two electrodes makes it impossible to identify the exact source of the θ-frequency. A number of reports registered a local generation of θ-oscillations in the cortical areas, including the enthorinal (Alonso and García-Austt, 1987) and cingulate (Leung and Borst, 1987) areas. Yet, the hippocampus is known as the primary source of θ-activity in the rodent brain (for a review, see Buzsáki, 2002). Such studies suggest a direct modulation of the cortical γ-oscillations by the hippocampal θ-activity, and appreciate the significant impact of the hippocampal θ-activity generated locally (Sirota et al., 2008). This modulation in rodents is likely to occur via volume conduction, as shown by Sirota and colleagues (Sirota et al., 2008).

The hippocampus receives little if any direct innervation from SCN (Watts et al., 1987; Morin et al., 1994; Abrahamson and Moore, 2001). Instead, the circadian signals may be relayed through the paraventricular nucleus of the thalamus, the lateral septum, or the bed nucleus of the stria terminalis (Morin et al., 1994). Several studies that utilized exotic rhythms, such as LD11/11, or constant light, to manipulate the circadian oscillator revealed an uncoupling between either the left and right SCN or their ventrolateral and dorsomedial parts, respectively (de la Iglesia et al., 2000, 2004). In some cases, SCN neurons become completely uncoupled from each other, yet keeping their own oscillations intact. Since the basal forebrain is crucial for hippocampal θ-activity generation, determining if changes in the circadian periodicity of the θ activity caused by an altered or absent inhibitory input from SCN to the neurons of the basal forebrain remains an appealing hypothesis to test.

Another consequence of rhythm shortening resulted in a highly elevated θ–γ PAC during wakefulness. θ–γ PAC was associated with active learning and memory retrieval (Canolty et al., 2006; Tort et al., 2009). In this respect, our findings are surprising, since studies performed on rodents (Devan et al., 2001; Loh et al., 2010; Karatsoreos et al., 2011) show a reduction in memory retrieval altered by the zeitgeber cycle. Moreover, a lesion of SCN restored the performance of hamsters with a preliminarily induced aperiodicity (Fernandez et al., 2014). From this perspective, future studies should address whether the observed changes in PAC are a consequence of the altered connectivity.

A potential confound of the current experimental design is that it does not separate the effects of a short light-dark cycle from that of short day or night. The solution for this could be to vary the lengths of either photo- or scotophase while control for the total length of the cycle. The analysis of EEG of rats (Franken et al., 1995), Siberian chipmunk (Dijk and Daan, 1989) and Djungarian hamster (Deboer and Tobler, 1996) housed under LD 8/16 and LD 16/8 asymmetric cycles show rather modest (< 10%) changes in total sleep time. In nocturnal species a short photoperiod caused re-distribution of time spent in NREM and REM sleep between short photophase and long scotophase, which is similar to our observations for LD11/11 and LD10.5/10.5 regimes. At the same time, the authors reported dampening of overall spectral power, including SWA, whereas we found reduction of PSD only for θ (7–9 Hz) frequency band during wakefulness, while the power of the other frequencies was either unchanged or elevated. Therefore, it is conceivable to conclude that while the re-distribution of sleep and wakefulness states may be caused by a short photophase, the changes of spectral composition we observed are an attribute of a short photoperiod.

In conclusion, the disruption of the circadian clock work, induced by a short light-dark regime causes significant changes of multiple parameters of brain physiology, ranging from the redistribution of vigilance states to alterations of PAC.

## Author contributions

SR planned and conducted the experiments, together with second author performed the surgery and EEG study, performed the data analysis and wrote the manuscript, JZ together with first author performed the surgery, set up and maintained the system for a long-term EEG/EMG recordings; participated in reviewing the manuscript, KG together with SR performed data analysis and participated in reviewing the manuscript, TP-H provided facility for animal experiments and participated in reviewing the manuscript, PP provided equipment and materials, including experimental animals; participated in reviewing the manuscript.

## Funding

The study was funded by the Academy of Finland (253416) and Sigrid Juselius Foundation to PP.

### Conflict of interest statement

The authors declare that the research was conducted in the absence of any commercial or financial relationships that could be construed as a potential conflict of interest.
